# Effects of hypothermically reduced plantar skin inputs on anticipatory and compensatory balance responses

**DOI:** 10.1186/s12868-016-0279-2

**Published:** 2016-06-29

**Authors:** Andresa M. C. Germano, Daniel Schmidt, Thomas L. Milani

**Affiliations:** Department of Human Locomotion, Institute of Human Movement Science and Health, Chemnitz University of Technology, Reichenhainer Straße 29a, 09126 Chemnitz, Germany

**Keywords:** Plantar hypothermia, CNS, Spinal cord, Dynamic balance, Sensitivity, anticipatory responses, Compensatory responses

## Abstract

**Background:**

Anticipatory and compensatory balance responses are used by the central nervous system (CNS) to preserve balance, hence they significantly contribute to the understanding of physiological mechanisms of postural control. It is well established that various sensory systems contribute to the regulation of balance. However, it is still unclear which role each individual sensory system (e.g. plantar mechanoreceptors) plays in balance regulation. This becomes also evident in various patient populations, for instance in diabetics with reduced plantar sensitivity. To investigate these sensory mechanisms, approaches like hypothermia to deliberately reduce plantar afferent input have been applied. But there are some limitations regarding hypothermic procedures in previous studies: Not only plantar aspects of the feet might be affected and maintaining the hypothermic effect during data collection. Therefore, the aim of the present study was to induce a permanent and controlled plantar hypothermia and to examine its effects on anticipatory and compensatory balance responses. We hypothesized deteriorations in anticipatory and compensatory balance responses as increased center of pressure excursions (COP) and electromyographic activity (EMG) in response to the hypothermic plantar procedure. 52 healthy and young subjects (23.6 ± 3.0 years) performed balance tests (unexpected perturbations). Subjects’ foot soles were exposed to three temperatures while standing upright: 25, 12 and 0 °C. COP and EMG were analyzed during two intervals of anticipatory and one interval of compensatory balance responses (intervals 0, 1 and 2, respectively).

**Results:**

Similar plantar temperatures confirmed the successful implementation of the thermal platform. No significant COP and EMG differences were found for the anticipatory responses (intervals 0 and 1) under the hyperthermia procedure. Parameters in interval 2 showed generally decreased values in response to cooling.

**Conclusion:**

No changes in anticipatory responses were found possibly due to sensory compensation processes of other intact afferents. Decreased compensatory responses may be interpreted as the additional balance threat, creating a more cautious behavior causing the CNS to generate a kind of over-compensatory behavior. Contrary to the expectations, there were different anticipatory and compensatory responses after reduced plantar inputs, thereby, revealing alterations in the organization of CNS inputs and outputs according to different task difficulties.

## Background

In daily life, we are confronted with various challenges regarding quasi-static and dynamic balance requirements. In both cases, the center of pressure (COP) needs to be corrected to keep or re-establish its position within the base of support. In terms of dynamic balance, postural strategies rely on two different mechanisms: anticipatory and compensatory responses [1, 2]. For example, when getting on a bus it is predictable that the bus will accelerate, causing a translational perturbation. To prepare the body for this upcoming external perturbation, anticipatory responses are accessed and induce pre muscle activation [2]. After the bus accelerates, causing the perturbation, reflexive compensatory reactions are generated resulting in muscular activation to re-establish balance [1, 3]. According to Santos et al. [2], the function of the anticipatory adjustments is to minimize the effect of the forthcoming body perturbations with some corrections while the function of the compensatory responses is to restore the balance after a perturbation has already occurred. In this way, both anticipatory and compensatory responses start with afferent information and they include activation or inhibition of muscles involved in postural control. All balance control responses are based on afferent inputs arising from visual, vestibular, proprioceptive and cutaneous systems [4, 5].

The relationship between cutaneous receptors in the foot sole and movement control is particularly well-known [6–8]. This relationship is apparent when considering patients suffering from peripheral neuropathy, which is most commonly a consequence of diabetes mellitus [9]. Those patients exhibit decreased plantar foot sensitivity [10] and reduced stability when performing dynamic balance tasks [11]. Since peripheral neuropathy reveals a multi-factorial character, affecting the peripheral nervous system and also resulting in joint immobility or foot ulcerations [12], it is unlikely that certain deteriorations in postural control are exclusively caused by reductions of foot sensitivity.

Therefore, to simulate only diminished cutaneous receptor activity, various methods are implemented, such as anesthesia, ischemia or cooling procedures [13–16]. Yasuda et al. [17] applied hypothermia to cool subjects’ feet using ice water for a duration of 20 min and subsequently performed double leg stabilometry. Subjects showed significant increases in sway area and sway velocity. Similarly, Magnusson et al. [18] also confirmed significant increases in sway velocity after hypothermic intervention at the feet during conditions with eyes open and closed. Another study demonstrated significantly greater center of pressure (COP) excursions after foot sole cooling while subjects performed double leg stances with closed eyes [19]. However, the same study found no significant differences when subjects’ eyes were open. In contrast, Billot et al. [20] showed that no increased COP excursions were evident after cooling plantar aspects of the feet. Furthermore, they explained these findings through greater EMG activity of the triceps surae muscles. Nurse and Nigg [21] analyzed walking parameters after plantar hypothermia affecting either forefoot or rearfoot areas. They demonstrated that peak pressures and pressure–time integrals were significantly lower in cooled areas, and they observed a shift of the COP towards more sensitive aspects of the foot. Due to these ambivalent findings, the exact role of plantar cutaneous input on balance control is still not fully understood.

Although the above-mentioned cooling procedures are frequently used as a tool to alter foot sensitivity, it is important to point out some limitations of these interventions. Firstly, when immersing the whole foot into ice water, for example, not only may cutaneous receptor activity be minimized, but joint receptors and muscle spindles may also be affected, as it was already proposed by Meyer et al. [8]. Secondly, after cooling and subsequent data collection, the feet may immediately reheat, for example while stepping on force platforms. As a consequence, the previous hypothermically diminished receptor activity might not be maintained at this level until the end of all trials. Finally, using ice water or ice exhibits difficulties regarding the control and maintenance of a determined temperature during the cooling process itself.

In view of this dilemma, the aim of the present study was to examine the effect of hypothermia of the foot sole during dynamic balance tests. Furthermore, to apply long-lasting and controlled plantar hypothermia and to avoid the involvement of other sensory systems, a self-developed customized thermal plate was integrated into the setup. This thermal plate allows foot soles to be cooled during data collection and enables measurements at intermediate temperatures, which may minimize unpleasant sensations for the subjects. After hypothermia, significant increases in both COP excursions and root mean square (RMS) of the electromyographic (EMG) signal were hypothesized.

## Methods

### Subjects

The experiments were performed on fifty-two healthy (26 female, 26 male) and injury-free subjects (mean ± SD 23.6 ± 3.0 years, 70.7 ± 13.0 kg, 1.7 ± 0.1 m). Only participants with no history of lower leg injury or lower-extremity pain for at least 6 months prior to testing and with no peripheral neuropathy or other related disorders were included in the study. In addition, none of the subjects were taking medication that could affect sensitivity or balance responses. All participants were informed about the purpose of this study and gave informed written consent. They were also instructed to interrupt the measurements if they experienced discomfort. All procedures were conducted according to the recommendations of the Declaration of Helsinki. The present study was approved (IfS Mil Mai Lastverteilungstypisierung 16052011) by the Ethics Committee of the Faculty of Behavioural and Social Sciences of the corresponding university.

### Apparatus

**Fig. 1:**
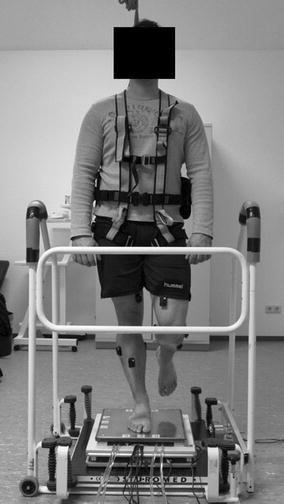
Picture of the apparatus used. Subjects stood with their dominant leg on top of the thermal plate which was mounted on top of the force platform. The force platform was then placed on top of the bottom platform of the Posturomed

A specialized apparatus was constructed to induce unexpected perturbations, to quantify balance ability, and to apply plantar hypothermia simultaneously (Fig. 1). The commercially available Posturomed device (Haider Bioswing GmbH, Germany) was used to induce unexpected horizontal perturbations [22]. Furthermore, this device is known to quantify dynamic postural control [22–27]. The Posturomed used in this study was equipped with an electro-magnet to attach the bottom platform 20 mm out of the neutral position [28]. After pressing a manual trigger, the bottom platform was released initiating the unexpected perturbation. Subsequently, the bottom platform swung horizontally until it reached the neutral position. Other versions of the Posturomed already include a lever-based provocation unit, which allows for similar perturbations [22]. Participants were secured using a safety belt and the handrail of the Posturomed was covered with insulation material to avoid injuries.

To quantify balance ability, a force plate (IMM Holding GmbH, Germany; sampling rate 1 kHz) was attached directly on top of the bottom platform. Finally, to induce plantar hypothermia, a self-developed customized thermal platform (Department of Human Locomotion of the corresponding university) was attached to the force plate. This thermal platform (temperature range 0–40 °C; resolution ±1 °C) consists of an upper aluminum plate which can be set to a desired temperature using the Peltier effect. Furthermore, to detect the reversal points of the oscillating bottom platform after triggering, a single axis accelerometer (ADXL78, Analog Devices Inc., USA; sampling rate 1 kHz) was included in the apparatus.

### Electromyography (EMG)

Wireless bipolar surface electrodes (Trigno™ Wireless, Delsys Inc., USA; DC-500 Hz, 160 dB/Dec.) were used to measure muscle activity of the following muscles of the dominant leg: M. tibialis anterior (TA), M. gastrocnemius medialis (GM) and M. fibularis (FIB). EMG data was pre amplified (1000×) and collected at a sampling rate of 1 kHz. EMG electrodes were positioned according to the recommendations of SENIAM [29]. Skin preparation included shaving, abrasion by sandpaper and cleaning with alcohol pads.

### Temperature

Before and after the experimental procedures, room temperature was monitored by a digital C28 type K thermocouple (Comark Instruments, U.K.) to maintain the room temperature between 23 ± 2 °C (EN ISO/IEC 17025) to avoid changes in measuring conditions. An infrared thermal camera FLIR E40bx (FLIR Systems Inc., USA) was used to measure the foot sole skin temperature at three anatomical locations (first/fifth metatarsal head (Met1/Met5) and heel) of the dominant foot. The anatomical locations were chosen since they are in direct contact with the ground when standing upright [30]. Experimental procedures were performed according to the standards of Protocols in Clinical Thermographic Imaging [31].

### Plantar hypothermia

Plantar hypothermia was induced by adjusting the thermal platform to three temperature stages in the following order: stage I (25 °C), stage II (12 °C) and stage III (0 °C). Stage I was the initial temperature with an acclimatization time of 3 min in which both feet were in total contact with the thermal platform at 25 °C. For stages II and III, the acclimatization time was 5 min at 12 °C and 10 min at 0 °C, respectively. The above-mentioned acclimatization times were sufficient to achieve and stabilize the desired temperature, avoiding extreme pain or discomfort, as shown in an unpublished pilot study. The temperature for stage II was chosen to examine possible effects regarding balance responses already occurring at the intermediate temperature. This was meaningful, since Schlee et al. [16] showed that plantar sensitivity is altered when varying plantar temperatures for 5–6 °C compared to the baseline.

Since measuring foot sensitivity is very time consuming, endangering the concentration of the participants, we did not include this issue in our protocol. However, in order to confirm decreased plantar sensitivity using the same hypothermic protocol with the thermal plate and temperature stages, we performed a pilot study: Plantar foot sensitivity (Met I) of ten young and healthy subjects (mean ± SD 25.2 ± 4.7 years, 70.4 ± 13.9 kg, 1.77 ± 0.11 m) was analyzed at 200 Hz. Cooling led to significantly decreased plantar sensitivity for all comparisons (α = 0.05): 25 versus 12, 25 versus 0 and 12 versus 0 °C (Table 1).

**Table 1:** Mean ± SD plantar temperatures (°C) and vibration perception thresholds (VPT, µm) for the first Metatarsal head (Met I) comparing all three temperature stages (25, 12, 0 °C)

	25 °C		12 °C		0 °C	
Met I	VPT (µm)	Temp (°C)	VPT (µm)	Temp (°C)	VPT (µm)	Temp (°C)
Mean ± SD	0.7 ± 0.4^∆,□^	25.1 ± 0.6^a,b^	1.8 ± 0.7^∆,○^	13.5 ± 1.0^a,c^	3.1 ± 1.8^□,○^	5.5 ± 1.8^b,c^

Significant differences are marked with superscripted symbols

Significant differences: VPT: ^∆^ *p* < 0.001; ^□^ *p* = 0.001; ^○^ *p* = 0.049; Temp: ^a^ *p* = 0.006; ^b^ *p* = 0.002; ^c^ *p* = 0.006

### Testing procedure

**Fig. 2:**
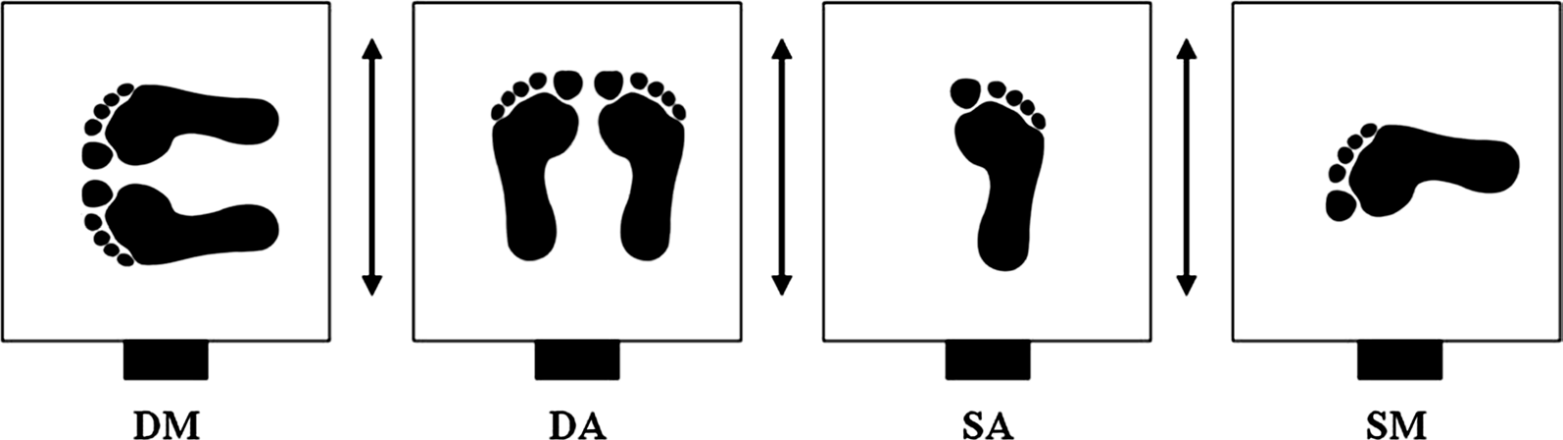
Illustration of all four balance conditions: double leg in medio-lateral (DM), double leg in anterior–posterior (DA), single leg in anterior–posterior (SA) and single leg in medio-lateral (ML) perturbation directions (*black arrows*). The electro-magnet is depicted as *solid black rectangles*

Prior to the measurements, all subjects performed several trials to become familiar with the apparatus. All trials were performed in four conditions which combined single (S) and double (D) leg stance with perturbations in anterior–posterior (A) and medio-lateral (M) directions: SA, SM, DA and DM (Fig. 2). Three trials were performed for each of the four conditions resulting in 12 trials per subject, which were executed in a randomized order. This series of 12 trials were then performed for each of the three temperature stages, resulting in a total of 36 trials for each subject. Plantar temperatures were measured before and after each series of 12 trials. During all measurements, subjects were instructed to look straight ahead, to keep their knees straightened but not locked, and to keep their upper limbs hanging down at each side of their body. Feet were positioned at the center of the thermal plate (marked with tape) for all tests. Furthermore, when conducting double leg stance conditions (DA, DM), subjects were asked to evenly distribute their body weight on both feet, keeping them shoulder width apart. While performing single leg stance conditions (SA, SM), subjects had to flex their contra-lateral lower limb backwards. Trials were deemed ineligible when subjects lost their concentration (e.g. talking), held on the handrail, or touched the ground with the contra-lateral foot. After the last temperature stage (0 °C), all participants’ feet were reheated using an infrared lamp radiator.

### Data collection and analysis

All force plate, EMG, and accelerometer data were synchronized using the manual trigger (onset of unexpected perturbation) and recorded by a routine written in LabView 8.0 (National Instruments Corp., USA). For further processing, all data was imported into the R program (The R Foundation for Statistical Computing, Austria). EMG data were offset-corrected and band-pass filtered (20–500 Hz; Butterworth 2nd order). Root mean square (RMS) of the measurements of the dominant leg was analyzed in this study. Center of pressure (COP) total excursions of the force plate data were low pass filtered (cutoff frequency 0.1, Butterworth 4th order). All data were analyzed for three time intervals in relation to the trigger (T_0_): −200 ms to T_0_ (interval 0), and two post trigger intervals: T_0_ to 90 ms post trigger (interval I) and 91–260 ms post trigger (interval II). Intervals I and II were assigned according to the first and second reversal points (90 and 260 ms, respectively) of the oscillating bottom-platform calculated over all subjects resulting in (mean ± SD) 90.0 ± 6.8 ms (reversal point 1) and 260.4 ± 17.0 ms (reversal point 2) which were determined using accelerometer data. Foot sole temperature data were analyzed using the software ThermaCAM™ Researcher Pro 2.8 SR-1 (FLIR Systems Inc., USA). Mean and standard deviation (SD) temperature values were calculated for each anatomical location (Met 1, Met 5, heel) of the dominant foot.

### Statistics

Means of the three trials were used to define the value of the analyzed parameters for each subject at each condition and temperature stage. Testing for normality was performed using the Shapiro–Wilk test (α = 0.05). EMG and COP data were analyzed using Friedman’s test followed by Wilcoxon-Test. Comparisons were performed between all three temperature stages for each condition. The level of significance was corrected due to the number of different temperature stages (n = 3) to α = 0.05/3 = 0.017.

## Results

### Temperature

Room temperature before and after data collection for each subject were (mean ± SD) 22.6 ± 0.6 and 23.3 ± 0.6 °C, respectively.

**Fig. 3:**
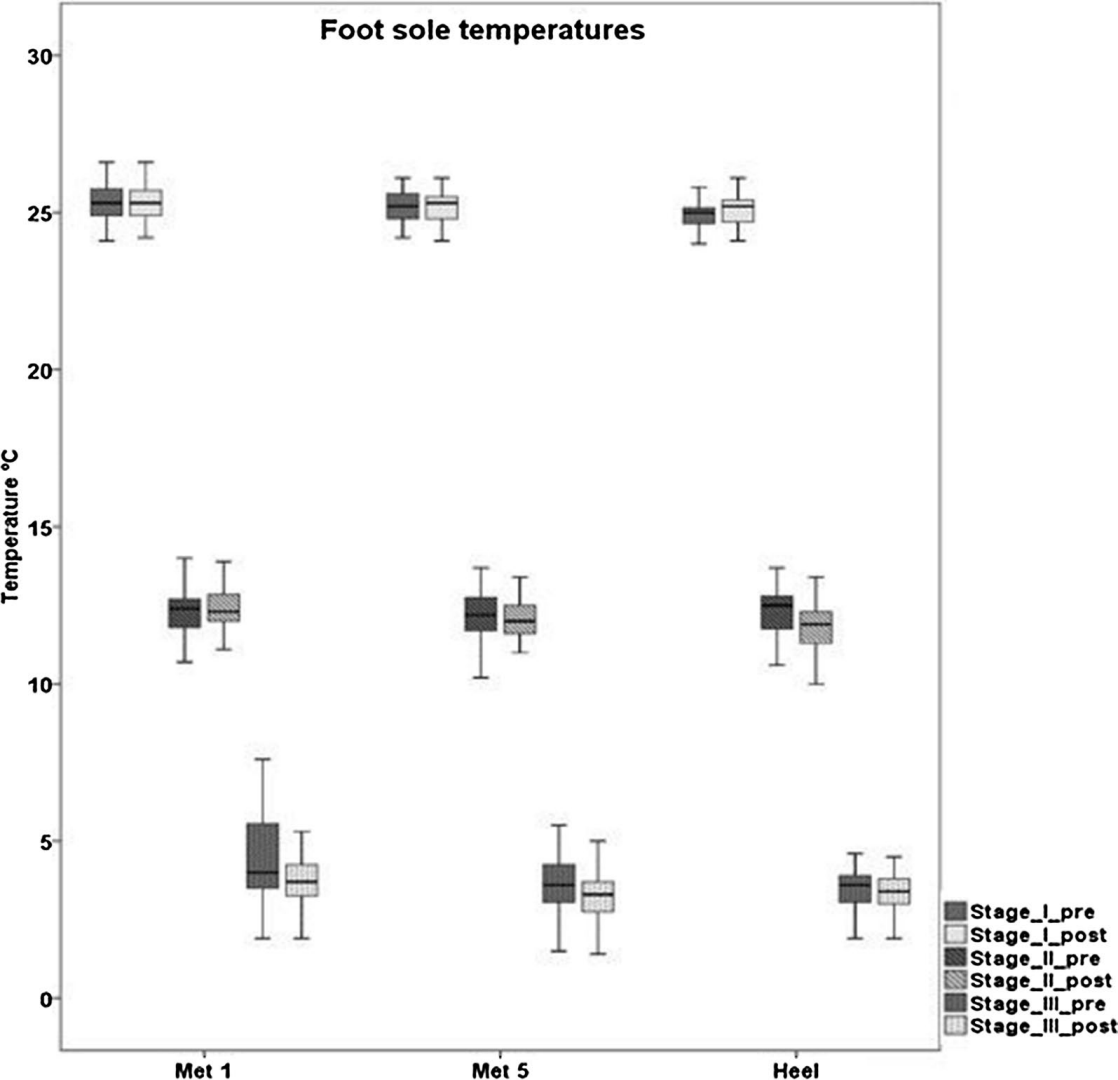
*Boxplots* of plantar foot temperatures of each anatomical location (Met 1, Met 5, Heel) and for each temperature stage (stage I, II, III) before and after the 12 trials

Figure 3 shows temperature data of the dominant foot sole (Met 1, Met 5 and heel) before and after the 12 trials for each temperature stage (25, 12 and 0 °C). No significant differences were detected within each temperature stage and between all anatomical locations. Comparing plantar temperatures for all three anatomical locations before and after the 12 trials, mean values ranged from: 25.0 to 25.4 °C for stage I, 11.9 to 12.4 °C for stage II and 3.2 to 4.8 °C for stage III.

### COP data

**Fig. 4:**
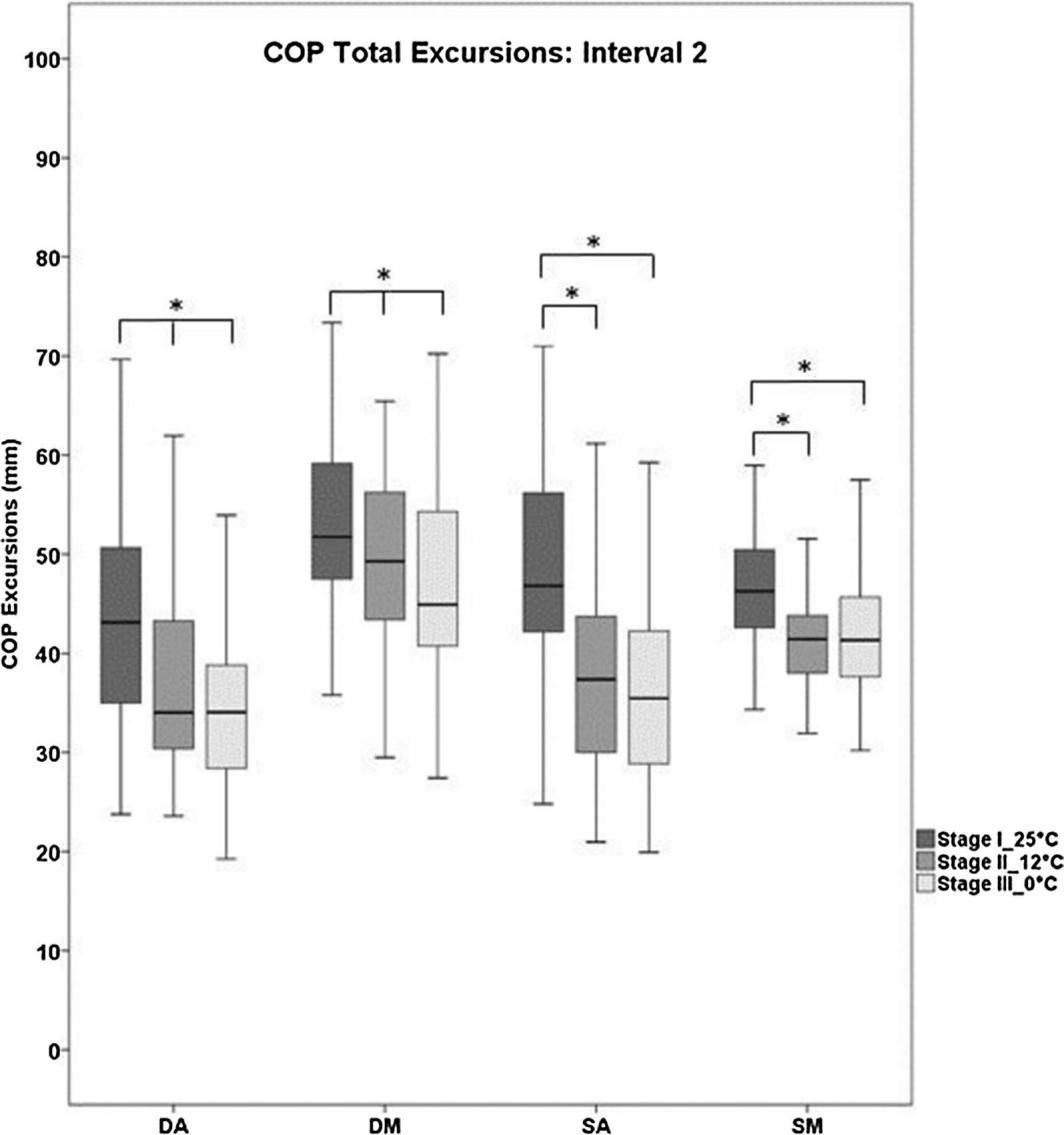
*Boxplots* of COP Total excursions for each condition (DA, DM, SA, SM) and each temperature stage for interval 2. Significant differences are marked with *asterisks* (*p < 0.017)

Table 2 and Fig. 4 exhibit data of COP total excursions for all four conditions and all three temperature stages. For interval 0, no significant differences were found between the temperature stages for all conditions. Similarly, interval 1 did not present significant differences between the three temperature stages in all balance conditions. In interval 2 and for double leg stance conditions, significantly smaller COP total excursions were revealed as plantar temperatures decreased. This was true for all temperature stages. In single leg stance conditions, significant differences were found when comparing stages I and II as well as stages I and III, with smaller excursions as temperatures decreased. No significant differences were detected when comparing stages II and III for SA and SM conditions.

**Table 2:** Mean ± SD COP total excursions for each condition (SA, SM, DA, DM) and temperature stage in intervals 0 and 1

	Interval 0			Interval 1			Interval 0			Interval 1		
Stages	25 °C	12 °C	0 °C	25 °C	12 °C	0 °C	25 °C	12 °C	0 °C	25 °C	12 °C	0 °C
	SA						SM					
COP Total (mm)	9.3 ± 2.9	9.0 ± 2.5	9.1 ± 3.4	29.1 ± 4.2	29.2 ± 3.7	29.5 ± 4.0	9.9 ± 3.0	9.0 ± 2.8	9.2 ± 2.9	33.3 ± 3.8	34.0 ± 3.8	33.3 ± 3.3
	DA						DM					
COP Total (mm)	5.8 ± 2.2	5.2 ± 1.8	5.6 ± 1.8	27.5 ± 4.4	27.5 ± 4.1	27.1 ± 3.9	5.3 ± 1.5	5.4 ± 1.7	5.8 ± 1.8	28.4 ± 3.8	28.8 ± 3.8	28.9 ± 3.8

*S* Single leg, *D* double leg, *M* medio-lateral, *A* anterior–posterior

### EMG data

**Fig. 5:**
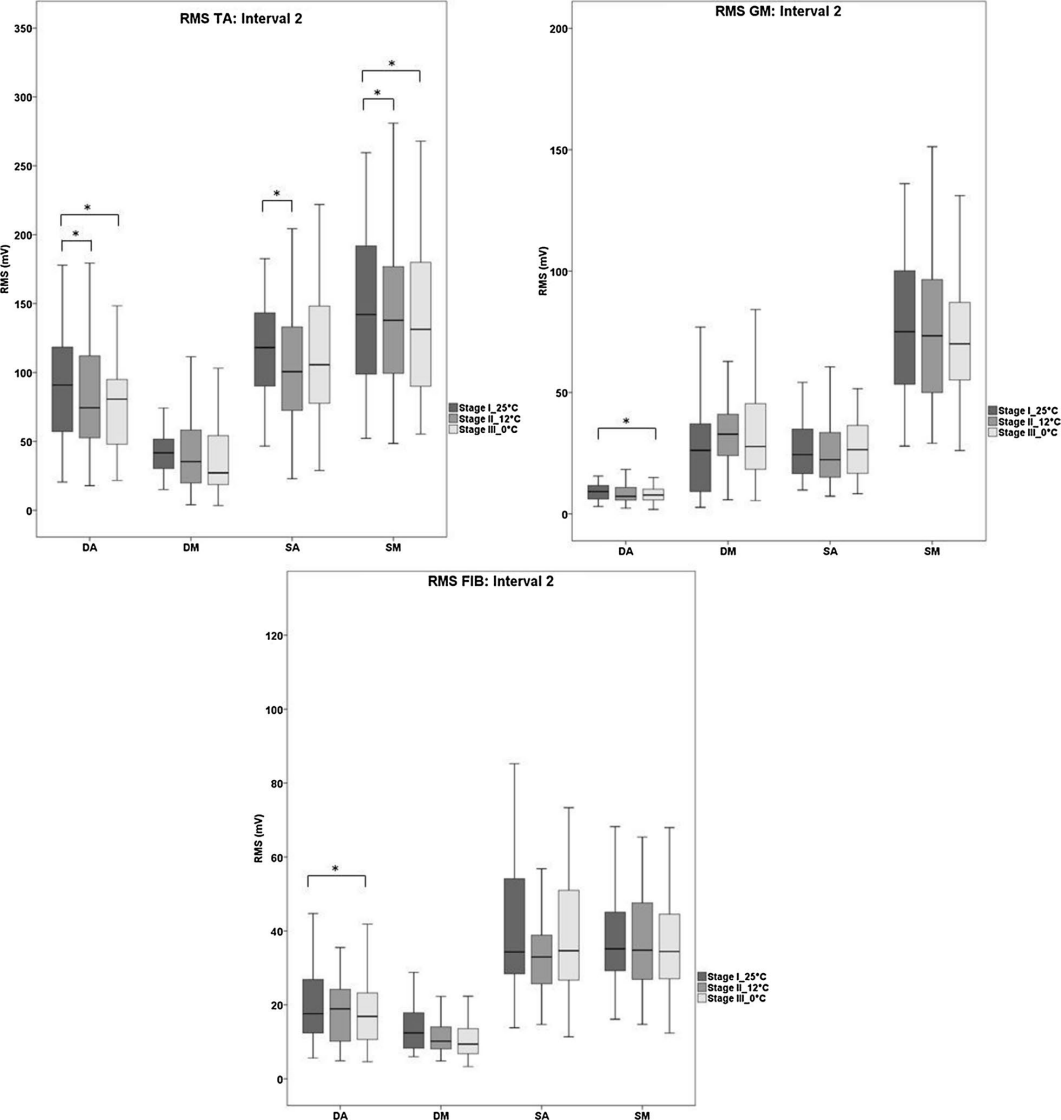
*Boxplots* of EMG values (RMS for TA, GM and FIB) for each condition (DA, DM, SA, SM) and each temperature stage for interval 2. Significant differences are marked with *asterisks* (*p < 0.017)

Intervals 0 and 1 did not show significant differences for all analyzed muscles (TA, GM and FIB) in all temperature stages and conditions (see Table 3). In interval 2, no significant differences were detected for DM in any analyzed muscles (see Fig. 5). For DA, significantly lower muscle activity was evident after cooling (stage III) compared to stage I for all analyzed muscles. Additionally, when comparing stage I with stage II, significantly decreased RMS values were found for TA after cooling. For both SA and SM, muscle activity of TA was significantly lower for stage II compared to stage I. Furthermore, for SM, the tibialis anterior muscle activity showed significantly smaller values in stage III when compared to stage I (see Fig. 5).

**Table 3:** Mean ± SD EMG values (RMS) for each condition (SA, SM, DA, DM) and temperature stage in intervals 0 and 1

	Interval 0			Interval 1			Interval 0			Interval 1		
Stages	25 °C	12 °C	0 °C	25 °C	12 °C	0 °C	25 °C	12 °C	0 °C	25 °C	12 °C	0 °C
	SA						SM					
TA	20.3 ± 13.3	16.8 ± 12.3	19.1 ± 12.0	16.2 ± 11.7	13.9 ± 11.5	16.7 ± 13.3	20.0 ± 13.8	19.3 ± 12.2	21.9 ± 15.1	16.2 ± 11.6	18.2 ± 15.6	18.8 ± 12.7
FIB	40.3 ± 26.7	40.5 ± 20.1	37.7 ± 19.1	39.9 ± 28.8	36.0 ± 21.9	34.8 ± 19.1	46.5 ± 27.8	47.8 ± 27.8	51.6 ± 30.4	47.1 ± 32.1	47.3 ± 29.9	49.4 ± 34.3
GM	37.7 ± 16.6	36.3 ± 13.0	37.5 ± 14.9	36.4 ± 18.7	37.4 ± 18.4	35.5 ± 15.2	35.9 ± 17.8	34.6 ± 17.2	35.5 ± 17.0	34.0 ± 17.1	33.6 ± 16.7	34.0 ± 17.7
	DA						DM					
TA	4.7 ± 5.1	7.3 ± 10.6	4.6 ± 6.3	4.4 ± 8.4	3.7 ± 4.3	4.0 ± 5.5	5.5 ± 5.2	5.1 ± 7.2	6.8 ± 10.1	5.4 ± 7.3	3.8 ± 5.8	6.6 ± 16.7
FIB	7.5 ± 8.6	5.8 ± 4.6	8.2 ± 7.1	5.6 ± 4.5	4.6 ± 3.1	5.6 ± 4.4	7.3 ± 7.4	5.4 ± 4.0	7.7 ± 7.8	6.7 ± 10.7	4.5 ± 3.0	6.2 ± 9.2
GM	9.7 ± 6.5	9.7 ± 6.9	7.8 ± 6.4	8.2 ± 6.3	7.7 ± 6.0	10.3 ± 14.6	7.4 ± 4.8	9.0 ± 6.8	6.7 ± 6.5	7.1 ± 7.1	6.7 ± 5.6	6.5 ± 6.7

*S* Single leg, *D* double leg, *M* medio-lateral, *A* anterior–posterior, *TA* M. tibialis anterior, *FIB* M. fibularis, *GM* M. gastrocnemius medialis

## Discussion

### Plantar temperatures

Plantar temperatures for all locations in stages I (25 °C) and II (12 °C) showed similar values to the temperatures predetermined by the thermal platform, before and after trials. For stage III (0 °C), plantar temperatures were also similar before and after trials. This shows that the temperature was kept constant throughout the entire balance measurement for each temperature stage. This outcome has large beneficial implications when combining plantar cooling procedures with various balance or gait measures.

It is well accepted that cooling the foot soles reduces plantar sensitivity [16, 19, 21], and it was also proved in our pilot study. However, previous studies examining the effects of plantar hypothermia did not ensure long-lasting cooling during trials, therefore, it is supposable that contact and/or friction to the warmer ground during standing possibly leads to re-warming, improving plantar sensitivity towards the baseline level. For example, a microneurographic study revealed a different finding: In one experiment, ice packets were placed over the receptive fields of a slowly adapting (SA) type II mechanoreceptor on the plantar foot for 15 min [32]. They showed that after 3 and 6 min of natural re-warming, receptor responses returned to 50 and 100 % of baseline firing, respectively. Due to those findings, we recommend ensuring permanent cooling during balance data collection to avoid possible improvements of plantar sensitivity. Another outcome of the present study was that in stage III the overall actual mean plantar temperature was higher (3.7 °C) than the platform temperature (0 °C). This effect is also observable in other studies and is known as cold-induced-vasodilatation to prevent cell death [33].

### Background on analyzed time intervals

To preserve balance, the central nervous system (CNS) uses responses which can be classified into anticipatory and compensatory adjustments [1, 2]. There are currently several different theories explaining the time windows in which those adjustments occur. To assess anticipatory responses in this study, a pre trigger interval (interval 0) was defined, similar to Santos et al. [2]. Additionally, they showed that anticipatory adjustments persist until after the trigger onset (T_0_) [2]. Therefore, interval 1 was chosen to analyze anticipatory adjustments. Since our setup induced horizontal translational perturbations at the subjects’ feet, in which muscle latencies occur at around 100 ms and active COP displacements at around 130 ms post perturbation [34–36], interval 2 was chosen to analyze compensatory responses.

### COP and EMG data

#### Anticipatory responses (intervals 0 and 1)

In intervals 0 and 1, no significant differences were found for all analyzed COP or EMG parameters in all conditions when reducing plantar temperatures. This finding was somewhat surprising, since many studies report reduced foot sensitivity due to cooling [16, 19, 21], which may result in a higher demand on postural activity. The findings of intervals 0 and 1 lead to the first two questions; a) what exact types of mechanoreceptors are being affected and/or b) whether or not these affected receptors are responsible for balance control in intervals 0 and 1. In this context, plantar mechanoreceptors vary considerably in their location, functionality, and frequency dependency. Slowly adapting (SA) mechanoreceptors (Merkel cells and Ruffini endings) are believed to contribute to postural regulation, specifically coding changes in COP parameters [14, 32]. Furthermore, SA receptors participate in responses to prolonged skin indentations [32], hence presumably playing an important role in intervals 0 and 1. Merkel cells are located superficially within the epidermis, whereas Ruffini endings are located slightly deeper within the dermis. Despite this, both SA receptors were shown to be effected by cooling procedures (2–20 min) [32]. This means there must be another explanation for why no significant differences were found in anticipatory COP or EMG responses, although plantar sensitivity was reduced. Regarding postural strategies, Winter et al. [37] reported that during quiet upright bipedal standing, balance control in the frontal plane is mainly achieved by hip and ankle muscles. Kelly et al. [38] demonstrated that during quiet upright standing with either one or two legs, intrinsic foot musculature shows high activation levels. Although intrinsic foot muscles are smaller than extrinsic foot muscles [39], they function as a unit [38]. Unfortunately, we did not quantify musculature activity associated with the hip strategy, nor did we measure intrinsic foot muscles, which could explain our findings.

Furthermore, there is another possible explanation for non-significant changes for intervals 0 and 1, which can be seen in the consequences of reducing specific sensory input. It is known that somatosensory, visual and vestibular information contribute to postural control. Peterka [40] argue that when healthy subjects stand on firm surfaces, they rely to 70 % on somatosensory, to 10 % on visual and to 20 % on vestibular input. However, the subsequent processing of that afferent information in the CNS does not follow a simple linear summation [41]. Ernst and Bülthoff [42] describe that senses arising from different modalities are merged in the brain to form a percept, which is achieved by sensory combination and sensory integration. Sensory combination describes the processing of various non-redundant sensory signals, while sensory integration occurs when signals are in the same unit and represent the same aspect of a specific environmental property, describing the processing of redundant signals. According to the concept of Ernst and Bülthoff [42], this can be transferred to our study: Non-redundant sensory signals may stem from visual, vestibular, proprioceptive and plantar skin sensitivity input. Those inputs need to be combined, resulting in redundant signals during postural tasks. That means, e.g. visual information needs to be combined with certain proprioceptive information to enable standardized body coordinates. These combined signals are then integrated to form coherent and robust percepts in the brain to maintain postural control. Interestingly, such sensory combinations have already been shown to exist in early stages of signal processing at the spinal cord level. For example, Lowrey and Bent [43] proved that there is a strong coupling between foot skin sensitivity and vestibular inputs, and that those two inputs influence muscle reflex activity. Another study also proved the relationship between plantar sensitivity and Achilles tendon reflex through induction of hypothermia [44]. Our findings suggest that for anticipatory responses during intervals 0 and 1, combination and integration processes may be responsible for a compensatory mechanism of the reduced plantar skin sensitivity. Hence, no deteriorated balance ability was found which would have been represented by significantly larger COP and/or EMG parameters, as hypothesized. The other intact sensory systems may then be reweighted to a higher degree to maintain balance control. Therefore, the assumption of a simple summation of sensory signals seems rather unlikely, as also affirmed by Mergner and Rosemeier [41]. It shall be acknowledged that a further explanation would be that cutaneous inputs from the foot soles simply play a minor or no role in balance regulation for intervals 0 and 1. However, we find this assumption rather unlikely since the contribution of plantar sensory inputs towards balance regulation is well proven and accepted [45, 46].

#### Compensatory responses (interval 2)

It is known that among others, also fast-adapting mechanoreceptors are responsible for such dynamic events, which provide information from the skin surface [32]. Meissner and Vater-Pacini corpuscles are fast-adapting mechanoreceptors, which are located in superficial and deeper skin layers, respectively. Lowrey et al. [32] demonstrated that after similar periods of cooling, mainly a reduced receptor firing rate was evident for both slow- and fast-adapting mechanoreceptors of plantar areas. Additionally, they showed that within cooling times of 2–5 min, there was no clear relationship between the receptor location (superficial or in deeper skin layers) and their firing responses. The results of our study support this outcome, since effects were already present when cooling for 5 min at 12 °C; whereas longer cooling periods augmented the effect of a reduced receptor firing response [32].

When comparing 25–12 °C in all conditions, significantly smaller COP total excursions were found with decreasing plantar temperatures. Furthermore, regarding RMS values over all muscles in all conditions, 9 out of 12 (75 %) values showed decreases as the foot sole was cooled, with three values showing significance for TA. When analyzing 25 versus 0 °C, all COP total excursions also showed significantly decreased values after hypothermia for all conditions. EMG activity presented similar behavior, except for two out of 12 comparisons (17 %). These results allowed us to conclude that effects were present in both hypothermic scenarios (25 vs. 12 and 25 vs. 0 °C).

When comparing 12 °C with 0 °C, COP total excursions exhibited significantly smaller values as plantar temperatures decreased, but only during bilateral stance. However, considering mean differences over all conditions for 25 versus 12 and 25 versus 0 °C, values were 4.5 and 6.1 mm, respectively, while mean differences for 12 versus 0 °C were only 1.6 mm. There were no significant differences for EMG activity for all muscles and all conditions. Interestingly, the same behavior for COP mean differences was observed for EMG mean differences. As mentioned above, effects were present, but when comparing 12 versus 0 °C it seems that this final temperature stage presents differences less relevant when considering EMG and COP data of this study. Hence, it can be presumed that the hypothermic treatment at an intermediate temperature (12 °C) would be enough to elicit effects (comparing 25 vs. 12 °C), which is an important finding for future studies. Although we did not assess the subjects’ perceived pain levels, they reported more unpleasant sensations during stage III (0 °C) compared to stage II (12 °C), indicating nociceptor activity. An interesting study conducted by Blouin et al. [47] found that with increasing stimulus temperatures, their subjects perceived pain, which deteriorated the postural control system by an interaction of nociceptor and Ia afferents at the spinal level. Therefore, it could be possible that nociceptive signals elicited in the lower temperature range may interact with α-motoneurons and, consequently, affect balance control in a negative way. This would further emphasize the importance of the effects already achieved at an intermediate temperature, as in our study.

A general finding was that—in the case of significant differences—both COP and EMG parameters always showed decreasing values as foot soles were cooled. Furthermore, most data with non-significant differences showed the same trend. When considering previous literature, decreased COP values were also found in the experiments performed by McKeon and Hertel [19]. Plantar aspects of the subjects feet were immersed into ice water for 10 min. Significant reductions of the COP area were detected, however, only with the eyes closed and in bilateral quasi-static stance conditions. In contrast, Magnusson et al. [18] found increasing parameters (body sway velocity) when the subjects´ feet were placed into ice water for 20 min, however, again in quasi-static conditions. Fukuchi et al. [48] found increased parameters for the SD of COP displacements and for COP velocity, but their subjects where immersed in cold water (approx. 11 °C) up to the umbilical level. After cooling the foot soles, Billot et al. [20] and Billot et al. [49] found no significant differences in COP excursions, but significantly increased muscular activity. Therefore, it becomes evident in previous studies that parameters show no clear behavior, but balance tests were of quasi-static nature which differs from our protocol in interval 2.

As already mentioned, our study demonstrated decreased parameters as plantar temperatures were reduced. Hence, our hypothesis of increased COP and EMG values has to be rejected. This finding was surprising, since decreased values are interpreted as improvements in balance [50–52].

The first explanation for the decreased values are possible learning effects, which seem plausible since we could not randomize the order of the temperature stages. This was because we did not estimate when plantar aspects would reach baseline temperature again after having been cooled. This process may impact the duration of our experiment, therefore, impairing the subjects´ concentration. Future studies should investigate the effects of different randomization approaches of temperature stages on sensitivity/balance parameters. Furthermore, even after sufficient time for re-warming, it is not known whether receptors would behave equally compared to the initial baseline measurement. In this regard, Kunesch et al. [53] demonstrated that even after the initial skin temperature was regained, some reduction of receptor sensitivity often persisted for a few minutes. Consequently, stage III was the very last stage and was therefore vulnerable for possible learning effects. Boeer et al. [54] confirmed that the Posturomed shows reproducible results, however, tests were performed in quasi-static balance conditions. In addition, in a previous study implementing a similar protocol with unexpected perturbations, we showed that the Posturomed exhibited good overall reliability [28]. Therefore, we can exclude possible learning effects as the explanation for the decreased COP and EMG parameters in the present study.

The most plausible explanation, therefore, lies in neurophysiological processes during interval 2, which differ from intervals 0 and 1. Although the subjects did not know when exactly the perturbation would occur, they knew that it would occur. Therefore, not only anticipatory adjustments were always present in intervals 0 and 1, but also compensatory responses in interval 2. However, afferent processes are different in interval 2: during dynamic balance tasks there were other still intact afferent channels responsive to a greater amount due to the perturbation (e.g. muscle spindles, joint receptors) and other impaired plantar channels (Meissner and Vater-Pacini corpuscles) contributing to postural control. Therefore, the processes of combination and integration from afferent inputs should be different and more complex than during the two previous intervals. Those aspects may lead to a more cautious behavior compared to intervals 0 and 1, since certain information regarding the base of support are missing or incomplete in addition to the higher demanding task. This would then be integrated into the actual postural motor program in the CNS. Efferent signals from the motor cortex innervate α-Motoneurons in the anterior horns of the spinal cord which drive musculature [55] via the medial and lateral corticospinal tract [56], hence controlling posture. This alpha-neuronal drive is modulated by convergent peripheral sensory input information via spinal inter-neurons with high degrees of “freedom” [57]. We assume that the more cautious behavior produced the necessity for a restricted area of base of support, consequently, preventing loss of balance. Since plantar inputs were impaired, other intact afferents were recruited and reweighted the combination and integration processes within the central nervous system. In the spinal cord, intact afferent information probably influence the actual motor program stemming from the cortex by high prioritized interneurons and intensify this precautious motor program. It is further known that sufficient threat towards posture must exist to induce adaptations in e.g. reflexes [58], which probably occurred in our protocol. Therefore, we presume that a kind of over-compensation due to the cautious behavior occurred which led to the reduced COP and EMG parameters found in this study.

We also expected that significant increases of muscle activity would also lead to significantly increased COP excursions after the hypothermic procedure. However, this relationship between COP and EMG was not always present in our results. This corresponds with Billot et al. [49], who also showed that significant differences in EMG parameters did not necessarily result in significant COP changes within the same trial. However, they investigated COP velocity. These outcomes can be explained in that EMG activity of the muscles we measured are not the only contributors towards COP displacements. In addition, there are delays regarding the onset of EMG activity and the subsequent onset of active COP displacements of approx. 20–40 ms [35]. Therefore, it is possible that the measured EMG and COP onset may not occur at the same time during this interval. Another possible explanation is that the measured muscle activities are not mainly responsible for COP changes. Nardone et al. [59] even argue that muscle strength does not play a major role in sway control when studying patients with peripheral neuropathy (Charcot–Marie–Tooth type 1A). As already mentioned, other intact inputs are integrated already at the spinal level, therefore contributing to balance control.

Finally, a further aspect of studies which reduce afferent information aim to simulate various diseases, like peripheral neuropathy. However, there are different mechanisms of the CNS, depending on whether sensory reductions are of acute or chronic origin. Our findings suggest that the acute reductions induced a more cautious behavior, in which a sudden reweighting of afferent inputs was necessary. On the other hand, in many chronic sensory reductions (diseases like diabetes mellitus) these reweighting processes are possibly accomplished more slowly, since sensory impairments also occur continually over long periods of time. Horak [60] even revealed that in patients with certain disorders (e.g. Alzheimer´s disease, peripheral vestibular loss, somatosensory loss from neuropathy), the ability of the CNS to quickly reweight sensory inputs is deteriorated. In agreement with this, Nardone et al. [59] examined patients with the Charcot-Marie-Tooth neuropathy type 1A who were divided into two groups. One group with a neuropathy score (NS) of 13 and the other group with a score <13. They found the sway area to be within a normal range for the less severely affected patients (NS < 13), but moderately elevated for the more severely affected (NS = 13). Therefore, we suggest (1) caution when interpreting COP data, because small excursions/low EMG activity does not necessarily correspond to improved balance abilities; and (2) reweighting and integration processes are strongly dependent on the type of disease, duration, and type of receptor impairment. Furthermore, the subject´s age and prior physical experience may play an important role within those processes. More studies are needed to clarify these relationships.

## Conclusion

The protocol using the thermal platform was successfully implemented since the plantar foot temperatures were similar pre and post trials. Anticipatory responses in intervals 0 and 1 revealed no significant differences as plantar temperatures were reduced. This might be explained by a compensation of the impaired afferent inputs by other intact inputs. In interval 2, compensatory responses of balance in all parameters showed generally decreased values as plantar temperatures were reduced. This is probably due to the balance threat caused by the unexpected perturbations, promoting a more cautious behavior which led to a kind of over-compensations of the CNS´ strategies regarding integration processes. It was further revealed that the intermediate hypothermic temperature stage II (12 °C) already elicited effects. Future studies which aim to induce hypothermia should investigate the influence of cold-induced pain on postural behavior. Further, reweighting processes of various inputs need to be examined more profoundly, whether intra and inter subject strategies are different, and if so, for what reasons. In this regard, analysis of EMG latencies could also be investigated to better understand dynamic balance responses. Finally, we recommend a cautious interpretation of data when discussing improved or deteriorated balance abilities, according to the particular subjects being examined.

## Abbreviations

COP: center of pressure
EMG: electromyographic activity
RMS: root mean square
TA: M. tibialis anterior
GM: M. gastrocnemius medialis
FIB: M. fibularis
SA: single leg stance in anterior–posterior direction
SM: single leg stance in medio-lateral direction
DA: double leg stance in anterior–posterior direction
DM: double leg stance in medio-lateral direction
CNS: central nervous system
